# Moisture control design has to respond to all relevant hygrothermal loads

**DOI:** 10.14324/111.444/ucloe.000037

**Published:** 2022-07-15

**Authors:** Hartwig Künzel, Mark Dewsbury

**Affiliations:** 1 Fraunhofer Institute for Building Physics, Holzkirchen, Germany; 2 University of Tasmania, Launceston, Australia

**Keywords:** moisture control design standard, hygrothermal simulation, vapour diffusion calculation, deemed to satisfy, component leaks

## Abstract

Moisture-related damage is still a formidable cost factor in the building sector. Besides installation deficiencies, moisture control design failures are the most frequent reasons for moisture problems. Therefore, adequate moisture control analysis has become the key for sustainable buildings. However, by only focusing on vapour diffusion other important moisture loads such as driving rain, construction moisture or air infiltration are mostly neglected. Therefore, international moisture control standards often refer to simulation models for more realistic analysis, leaving many practitioners wondering how to handle these tools. To overcome this dilemma, the updated German moisture control standard has introduced a three-pathway approach for design evaluation: first, deemed to satisfy list, second, restricted Glaser calculation and third, fully fledged hygrothermal simulation. The third pathway includes the option to account for small leaks or imperfections in building envelope components. Guidelines in other countries are also embracing similar moisture control approaches which gives hope for more durable and sustainable building design. To reach this aim, moisture control should also become an integral part of the design process instead of a secondary chore.

## Introduction/background

Moisture in the building structure impairs thermal performance and accelerates ageing and degradation. Therefore, moisture control has always been an issue for architects and engineers. Despite an improvement in construction quality, moisture problems have not diminished accordingly. This may also be due to increasing energy efficiency requirements. More insulation and better airtightness have resulted in lower temperatures at the exterior layers of the building envelope and higher indoor humidity. Thus, the risk of interstitial condensation has become more prominent while the drying potential has stayed the same or has decreased. The high number of moisture-related building failures also demonstrates that traditional condensation control is not sufficient to solve the current problems. Additionally, the concentrated and intense rain events, or natural disaster events causing torrential rain and flooding are becoming more frequent, which means more risk for carbon sequestering but often moisture susceptible renewable materials used in the external envelope of new buildings. However, adequate moisture control design can help to prevent problems even in the most energy efficient and sustainable structures. The necessary design tools are there, but they must be applied in the right way. While it is fair to assume that a building has been erected according to best practice, a perfect seal against water, vapour or air entry is difficult to achieve. Therefore, the consideration of imperfections should be part of moisture control assessments.

In the past, most design and construction practitioners utilised steady-state vapour diffusion calculations, often called dewpoint or ‘Glaser’ calculations for moisture control analysis. However, due to numerous simplifications the results of these calculations may be misleading especially when capillary flow or moisture storage have an impact. This penalizes materials with moisture storage capacity, such as bio-based insulation materials, because it assumes formation of condensation where in real life only the sorption moisture content is slightly increased. There are actually many more drawbacks from employing dewpoint calculations for moisture control design which have turned the focus to complex numerical simulation. Transient hygrothermal simulation offers an opportunity to meet the current challenges if the underlying models allow for the fact that no building component is perfect and envelope leaks and other flaws are always built-in or may eventually occur during the building’s service life. New approaches have been developed to account for rainwater penetration and indoor air infiltration. This paper discusses the moisture loads acting on the building envelope components and summarises the approaches in some current moisture control standards and guidelines.

## Moisture loads

**Figure 1:**
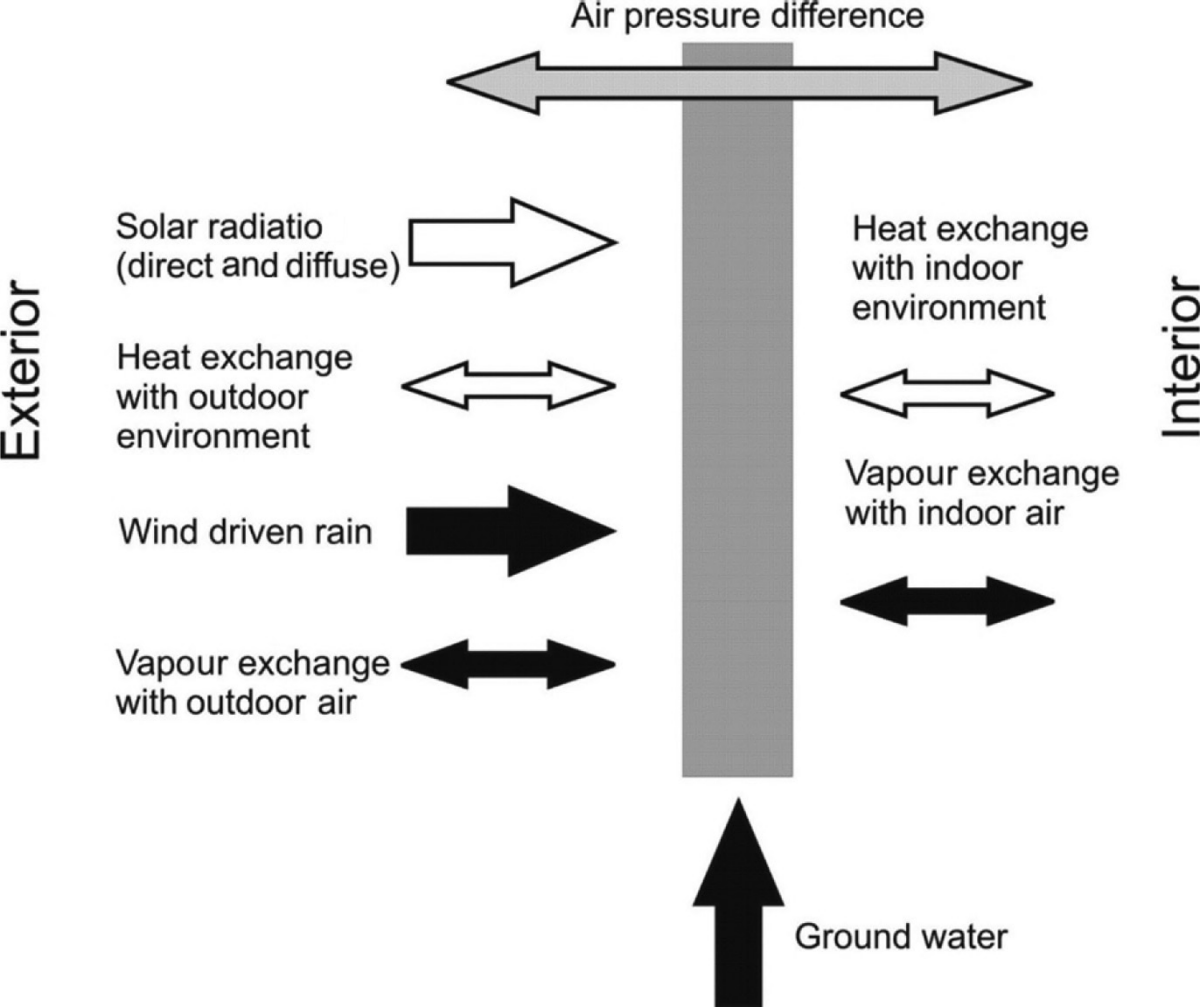
Schematic representation of the moisture loads acting on the building envelope caused by dynamic hygrothermal parameters and their alternating diurnal or seasonal directions according to the *ASHRAE Handbook of Fundamentals* [1].

The main functions of a building envelope are to protect the enclosed space against natural weather and to provide an adequate indoor environmental quality for human occupation. In Fig. 1 the moisture loads, or better hygrothermal loads, acting on building envelope components are represented schematically for the example of an external wall. Generally, they show considerable daily and annual variations at the exterior surface which are propagated only to a minor extent to the interior surface. During daytime the exterior surface heats up by conduction and solar radiation, which leads to evaporation of moisture from the surface layer. Around sunset when the shortwave solar radiation ceases the longwave (infrared) emission may lead to an overcooling (cooling down below ambient air temperature) of highly insulated wall systems and condensation may occur on, and within, the façade system. However, if the building envelope is exposed to driving rain the water load is generally more important. Often, several hygrothermal load cycles are overlapping such as summer/winter, day/night and rain/sun. Therefore, a precise analysis of the expected loads should be completed before designing a new building or changing the envelope of an existing construction.

### Outdoor air conditions

Ambient temperature- and humidity-caused vapour pressure are the continuously changing boundary conditions permanently acting on both sides of the building envelope. The exterior conditions depend on the climate and the local environment. Practical experience (in Europe) tells us that large temperature differences over the building envelope result in large partial vapour pressure differences and hence considerable vapour diffusion/condensation loads. Therefore, we expect surface and interstitial condensation to be rather severe when it is cold outside. Consequently, we place our vapour control layers (a more precise expression for vapour barrier or vapour retarder) on the inward side of the insulation.

**Figure 2:**
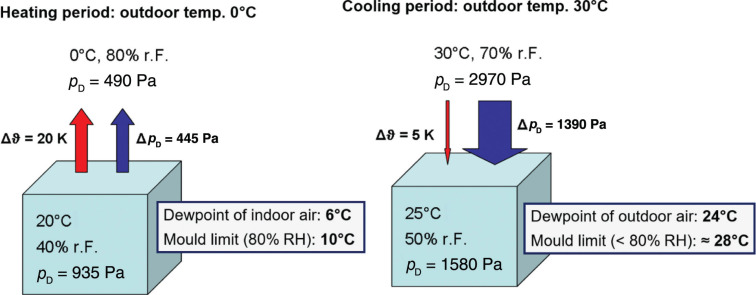
Typical temperature and vapour pressure gradients between outdoor and indoor conditions during the heating season in cold and moderate climates (left) and the cooling season in warm and humid climates (right).

What about more temperate or hot and humid climates? While average temperature differences of 20 K between indoors and outdoors in winter are rather common in cold and moderate climate zones, the typical temperature differences in temperate climate zones can vary significantly between the summer and winter seasons, whilst in the tropics they are much smaller, for example, 5 K (30°C outdoors and 25°C indoors). In the case of the tropics, this appears to be rather benign, and we would not expect any condensation or mould growth problems to occur. Alas, this is not true, because the saturation vapour pressure increases exponentially with rising ambient temperature. The consequences may be demonstrated by the example in Fig. 2. On the left-hand side, typical daily average indoor and outdoor climate conditions are listed together with the resulting temperature and vapour pressure differences over the building envelope. Comparing the outdoor climate with the dewpoint of the indoor air and the limit temperature for mould growth [i.e., the temperature where the indoor air would reach 80% relative humidity (RH)], proves that indoor air may cause condensation and possibly also mould growth in well-insulated components. The risks of this happening increase if the indoor humidity finds a shortcut to the exterior component layers, for example, by convection or vapour diffusion through gaps in the construction. The situation on the right-hand side is representative for the cooling period in a tropical climate. While the daily average temperature difference is only a quarter of that in a heating climate, the vapour pressure difference is threefold. As the dewpoint of the outdoor air is lower than the indoor temperature, condensation in the building envelope will not occur, unless the room is conditioned below 24°C – which may happen when the air conditioner (AC) is controlled by a humidistat. However, even if there is no condensation, the conditions for mould growth may still be favourable. The limit temperature of a material layer for mould growth to occur is approximately 28°C. As spore germination occurs more rapidly when the temperature is well above 10°C, the risk of mould growth cannot be ruled out if outdoor humidity is not prevented from entering the building envelope.

### Wind-driven rain

Apart from arid climate zones, it has been found that the most important exterior moisture load for external walls is often wind-driven rain. Similar to solar radiation, driving rain is a directional parameter and often has a dominant orientation. The dominant orientation depends on the latitude (e.g., west in Europe, east on the Canary Islands) and it may also show a seasonal variation (e.g., East Asia). The driving rain load also depends on the exposure (e.g., landscape, surrounding buildings) and the height of the building. While the main load hits the upper part of the façade, the lower part often shows more water-related damage, because water draining down, coupled with slower evaporation (less wind and incident solar radiation) keeps the bottom wet for some time. However, the worst-case scenario represents rainwater penetration through joints and connections. Large-scale damage of lightweight walls with external wall insulation systems also called exterior insulation and finish systems (EIFS) in the United States (US) were reported around the turn of the century. A comprehensive literature review, analysing more than 10 investigations undertaken on several buildings [2], came to conclusions that the main reasons for moisture damage were leaks around windows and joints where rainwater penetrated beneath the insulation layer.

**Figure 3:**
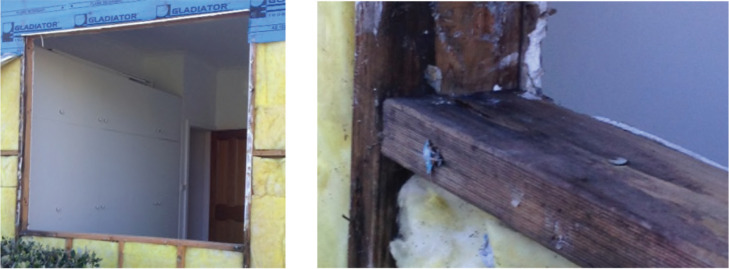
Example of moisture ingress causing degradation of timber framing in Australian residential construction.

In Australia, due to the more temperate climates and the smaller requirement for external wall insulation, most buildings include insulation within a 90 mm framing system. Since 2007, many of these have included a pliable membrane on the outside of the insulated frame. Except for clay brick veneer, most cladding systems are generally directly fixed to the timber or steel frame, resulting in no drainage or vapour cavity between the cladding system and the pliable membrane. As in the US example discussed above, recent research has identified built fabric degradation caused by both moisture ingress through gaps and breaks in the pliable membrane and moisture accumulation caused by the use of climatically inappropriate pliable membranes and thermal bridging between direct fixed cladding and pliable membranes [3]. An example of moisture ingress is shown in Fig. 3.

**Figure 4:**
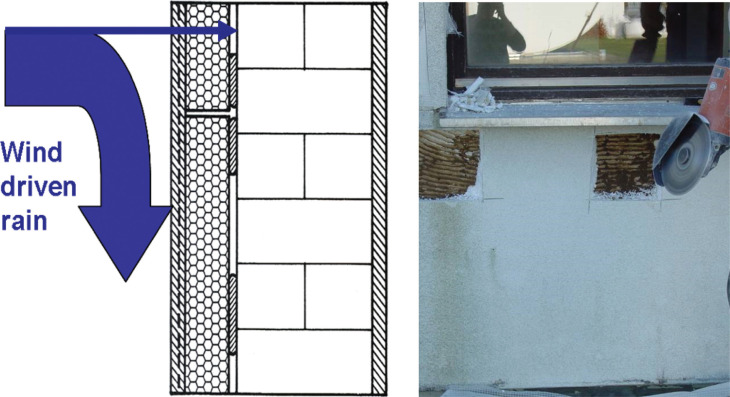
Exposed wall example showing the most likely deposit location for rainwater penetrating through leaks at windows and other connections of masonry with an external wall insulation system (left). The result of such leaks can be observed in the photo on the right-hand side. While probing the external insulation of a masonry wall underneath west-oriented windows, the living space of ants appeared behind the EPS slabs, demonstrating that the ants appreciate the warm and moist environment in the gap between the EPS insulation and the masonry.

In Central Europe where these systems [mostly called external thermal insulation and composite systems (ETICS) directly translated from the German Wärmedämmverbundsystem] are also very common, only a few damage cases had been reported at the time when the failure of similar systems became notorious in North America. On the contrary, these systems had a rather good track record and performed mostly very well over several decades [4]. American experts visited Germany to explore why the German envelope systems performed better than the US envelope systems. The analysis identified that the details around windows were not that different. Samples taken from underneath west-oriented windows in masonry walls covered with expanded polystyrene (EPS) external wall insulation systems found moisture behind and in the insulation layer (up to approx. 10% in volume). In one case ants were living in the moist and warm gap between masonry and insulation layers (Fig. 4). The reason for the difference in track record between the German, Australian and North American systems was found to lie in the load bearing structure. While timber frame structures are rather susceptible to moisture, masonry and concrete can absorb large amounts of water before any damage becomes apparent. This could be confirmed by performing hygrothermal simulations including intentional rainwater leakage during driving rain spells [5].

### Solar vapour drive

Solar vapour drive represents an exterior moisture load that is often underestimated by architects and engineers. This effect is the result of rainwater being absorbed by reservoir cladding, such as brick veneer or external rendering systems. If the sun heats up the wet cladding, the water will dry out in both directions, that is, a large part of it will also move into the wall. Depending on the construction, this may cause condensation and potential damage in the case of moisture susceptible materials. This phenomenon has been studied in the ASHRAE Research Report [6] and is also described in the ASHRAE Handbook [1]. It happens mostly in summertime and is caused by vapour diffusion from the wet cladding towards the material layers behind it. It is not restricted to walls but may also happen in pitched roof constructions if roofing tiles are water absorbing and vapour permeable. It is obvious that this effect cannot be captured by steady-state diffusion calculation methods as it is highly dynamic and requires a combination of driving rain events and sunny spells. The impact of solar vapour drive may be alleviated by the inclusion of cladding ventilation [7].

However, while cavity ventilation may be beneficial to prevent damage to timber structures due to solar vapour drive, it is mostly unnecessary to protect masonry cavity walls [8]. The impact of solar vapour drive can easily be buffered by the masonry, which means the moisture arriving at the surface of the load nearing wall will be absorbed without harming the masonry and released again when conditions change. In terms of energy consumption, ventilating such cavity walls is counterproductive. Considering all aspects, ventilating the cavity of masonry walls is not the preferred option. If desired, they can be drained or vented (cavity without top vents) but in most cases this may be unnecessary, for example, retrofitting walls by installing appropriate loose fill insulation in the entire cavity. Solar vapour drive happens in all exposed rainwater absorbing building components including solid wall structures. However, in solid masonry walls it usually competes with liquid flow, driven by capillary suction. As soon as the gradients of vapour pressure and suction pressure become opposed to each other, the total moisture flux through the wall will be reduced. In general, the remaining vapour flux towards the interior wall surface can be safely extracted by building ventilation. Water stains discovered at the interior surface of solid masonry walls are normally caused by liquid rainwater penetration rather than solar vapour drive. This may be prevented by improving the exterior driving rain protection, for example, by reducing the water absorption capacity of the external surface and/or by ensuring the airtightness of the wall structure if necessary. Masonry walls without render and plaster are very air permeable, and rainwater may be pushed through the wall due to air pressure differentials.

### Indoor climate

The indoor climate conditions depend on the purpose and occupation of the building. They are controlled to keep the interior space comfortable for human beings and/or suitable for furnishings or artefacts. However, for the building envelope they represent an important load that can be more severe than the exterior load especially when the indoor moisture generation is high. In most commercial buildings, temperature and humidity are controlled by ducted heating, ventilation and air conditioning (HVAC) systems whose set points are usually well defined. The interior conditions in residential buildings are influenced by the behaviour of the occupants. In an average household (four persons) up to 10 litres of water are created every day [9]. This moisture must be removed by ventilation or air conditioning in order to ensure comfortable and hygienic conditions. Special buildings like churches or heritage constructions may only be occupied temporarily, which means that the interior conditions can vary between normal indoor conditions and average outdoor air conditions with a phase shift. Depending on the kind of heating system, for example, local infrared or convective heating, a non-uniform indoor temperature may result, which raises the risk of surface condensation at cold surfaces.

The indoor humidity may enter the building envelope by vapour diffusion during the heating period. Therefore, lightweight constructions in cold and moderate climates are often equipped with an interior vapour control layer. However, the effectiveness of this layer is often compromised by the methods different elements connect, which leads to indoor humidity entering the envelope by air infiltration (slow convective airflow), especially in winter time when buoyancy effects cause pressure differentials over the upper envelope components of heated buildings. Damage records of non-ventilated flat timber roofs have proven that this kind of air infiltration is a major source of failure. Even roofs sealed according to best practice, with their airtightness checked by the blower door test, may be susceptible to moisture damage, because there are some imperfections. If small airflow leakages cannot be avoided, they must be accounted for in moisture control standards, which will also be briefly explained below.

### Construction moisture

One moisture load, the so-called construction moisture, is often forgotten. In Europe, building damage resulting from construction moisture has become more frequent because tight construction schedules leave little time for building materials to dry out. Construction moisture is either delivered with the building products or it is absorbed during the construction process. Cast in place concrete, autoclaved aerated concrete (AAC), calcium silicate brick (CSB) and green sawn timber are examples of materials that contain a lot of moisture when delivered. Stucco, mortar, clay brick and concrete blocks are examples of materials that are either mixed or brought into contact with water at the construction site. Furthermore, most building materials may absorb precipitation or groundwater when left unprotected before the enclosure of the building.

**Figure 5:**
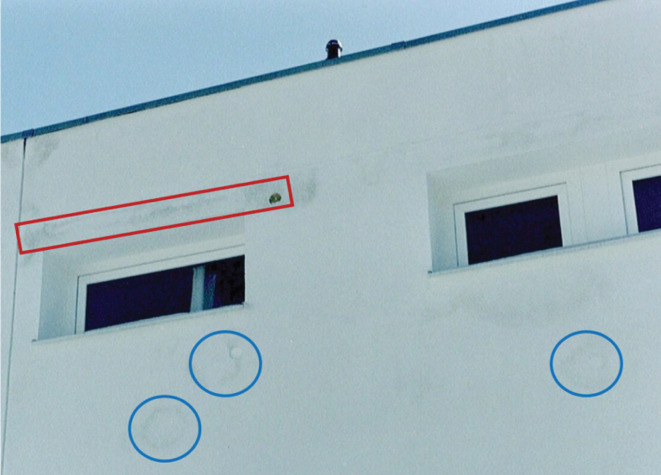
External wall insulation system on concrete. The construction moisture of the concrete caused staining on the rendered façade due to vapour diffusion from the wall through permeable zones of the EPS insulation layer. The vapour permeable zones above the windows (highlighted by a red rectangle) consist of mineral wool bars installed for fire safety; the round stains below the windows (indicated by blue circles) are caused by defective joints between the poorly installed EPS slabs [10].

Construction moisture may cause damage that remains undetected for quite some time. Moisture from masonry can migrate into the roof structure, causing hidden mould growth or corrosion in unventilated assemblies. Construction moisture drying indoors needs to be ventilated out of the building or extracted by adsorption drying respectively air conditioning to prevent elevated indoor humidity. Otherwise, delamination of ceiling plaster, warping of wooden floor panels, surface mould growth, etc. may occur. Construction moisture drying outwards may cause staining or frost damage on external rendering or coating systems [10, 11]. Figure 5 depicts the façade of a concrete wall with external wall insulation. The main part of the insulation layer consists of EPS slabs which have a low vapour permeability. However, due to German fire safety regulations the window heads must be insulated by mineral wool insulation. Having a very high vapour permeability, the construction moisture is funnelled through these zones leading to moisture staining on the external render mostly caused by microbial growth.

Construction moisture also reduces the thermal resistance of building envelope components and requires heat to evaporate. Sometimes, this may increase the energy consumption up to twice the normal rate during the first heating period. In extreme cases, additional heaters must be provided temporarily to ensure fast dry out to safeguard comfortable and hygienic indoor conditions.

### Rising damp

Moisture from the ground can be a problem for timber structures with crawl spaces. In contrast to concrete slabs on ground or concrete floor panels over crawl spaces, timber floors should not be exposed to the high humidity that may prevail in a crawl space. Vapour barriers and insulation on the ground or below the floor as well as controlled ventilation may help to solve this issue. More difficult to solve is rising damp in old masonry buildings. If water from the ground is responsible for the observed phenomenon, water repellent borehole injections may reduce the flow of water wicked from the ground. More effective but also more expensive are wall saw and seal technologies. However, what appears to be rising damp may also be summer condensation or hygroscopic moisture. Old buildings have often been exposed to different salts, for example, de-icing salt or nitrates originating from animals being kept in the building or the walls being exposed to sewage in the streets. When the salts are absorbed by the masonry, they increase the hygroscopicity and the vapour diffusion resistance of the wall surface [12]. Thus, the wall surface is moist and may even look wet, without any water being wicked from the ground. As the salt content decreases from bottom to top, this phenomenon can easily be mistaken for real rising damp. Yet, the solutions are very different. Remedial measures include extracting the salts by sacrificial plasters or by so-called renovation plaster systems (Sanierputz in German). The latter are composed of two layers, a capillary active layer wicking the salt solution out of the masonry, and a second water repellent layer with large pores in which the crystallising salt is retained and prevented from efflorescing at the surface. Rising damp is difficult to reproduce by hygrothermal simulation because the moisture boundary conditions in the ground are generally unknown. Additionally, mortar joints represent a resistance to the capillary flux. Therefore, the results of rising damp simulation may be of limited accuracy, yet they can help to compare the performance of different options.

In summary, the impact of moisture on building envelope components is not restricted to vapour diffusion from the interior spaces. More importantly, the impact is often multi-dimensional, highly dynamic and escapes simple steady-state assessment methods. In the past moisture problems were mostly associated with interstitial condensation during the cold season. However, rainwater penetration and solar vapour drive, which dominate in summer, may also cause severe problems. Therefore, we need tools to evaluate moisture risks that are beyond the scope of simple interstitial condensation calculations. Furthermore, there is consensus in building science that mould growth may appear at high humidity before interstitial condensation occurs. This problem will not be detected if the absence of interstitial condensation is the sole criterion for safe construction assemblies. Therefore, the following section describes and explains more sophisticated evaluation methods which have been or are about to be adopted in moisture control design standards and guidelines in Europe, North America and Australia.

However, these tools and their underlying physical models are subject to restrictions. The effects of salts or other pollutants on moisture transport properties are only partly known. The same applies for the impact of material ageing and degradation. Calculation results depend on reliable input data. Selecting appropriate data and interpreting the results correctly remains a challenge. Therefore, we need standards and guidelines as well as common sense and experience to design damage-free and durable building envelope systems.

## Considering construction imperfections in the evaluation of moisture risk

As already mentioned, building envelope systems are never perfect. Small leaks are unavoidable even in best practice cases. This issue is not new, but it represents a formidable challenge to all efforts to develop effective moisture control design guidance. Heinz Trechsel, editor of ASTM Manual 40, *Moisture Analysis and Condensation Control in Building Envelopes* [13], published in 2001, explained in the first chapter that there has been a formidable progress since the publication of the ASTM manual 18 in 1994. He clarifies that there are different types of computer models to calculate moisture transfer in building envelope assemblies of which the simpler ones are already employed in building practice. The more sophisticated models are already capable to account for air infiltration and rainwater leakage. However, in 2001 they were mostly used for research purposes because moisture entries by rainwater leakage or indoor air convection through imperfections in the building assembly have been regarded as multi-dimensional phenomena that required deep insight into the envelope details and complex mathematical models to account for the multifaceted moisture pathways.

Trechsel concludes

*“Although models that include air infiltration and rainwater leakage are excellent research tools, models that do not include these transport mechanisms are still most useful for the designer/practitioner provided that their limitations are recognized and proper precautions are taken to reduce or eliminate air infiltration and water leakage. The use of moisture analysis alone does not guarantee moisture-resistant buildings. Careful detailing of joints and the use and proper application of sealants and other materials are necessary. The issues of field installation and field quality control, mentioned above, must be addressed adequately by the designer and specification writer.”*

This indicates that 20 years ago building scientists were already well aware of the problem of construction imperfections and the challenge this poses to realistic moisture control analysis, but there was also hope that better detailing, proper installation and quality control would eventually solve this problem. Today, we have become somewhat less optimistic, because it appears to be impossible to make building envelope components waterproof and completely airtight under all circumstances, including over their whole service life. Therefore, the old paradigm of keeping constructions dry by installing water, vapour and air barriers has been succeeded by a new concept that stresses the importance of moisture tolerance by limiting moisture entry and maximising drying potential. Consequently, we now talk about water, vapour and air control layers.

### Driving rain leakage

Rainwater penetration should be prevented by all means. However, this is easier said than done, as perfect and durable sealing especially around windows is very difficult to achieve. Other connections may also provide entry to rainwater, such as roof–wall connections, penetrating pipes or ducts. Therefore, the so-called face sealing method should be supplemented or replaced by approaches that rely on drainage and/or ventilation. An example for face sealing methods are external wall insulation systems (EWIS), which have demonstrated time and again that rainwater penetration may cause severe problems as already mentioned above. The current trend to replace EPS by wood, hemp or other plant fibre insulation in EWIS can only be sustained if some kind of drainage is provided at vulnerable connections, unless the drying potential by vapour diffusion far exceeds the wetting potential by rainwater penetration. To determine the magnitude of the wetting potential requires reliable models that account for rainwater penetration which is representative for best practice installations. Defining number and size of potential leaks for different façade systems is obviously challenging. However, any reasonable assumption for rainwater penetration is better than nothing, that is, assuming that a perfect seal against driving rain exists. The American moisture control standard ANSI/ASHRAE 160 [9] has been the first guideline to address this challenge in its first version in 2009. It proposed the consideration of small rainwater leaks through the exterior finish, which may result from gaps or cracks at joints and connections, by stating:

*In the absence of specific fullscale test methods and data for the as-built exterior wall system being considered, the default value for water penetration through the exterior surface shall be 1% of the water reaching that exterior surface. The deposit site for the water shall be the exterior surface of the water-resistive barrier. If a water-resistive barrier is not provided, then the deposit site shall be described and a technical rationale for its selection shall be provided.*

In the case of EWIS on load bearing masonry walls the rainwater deposit site is likely to be the surface of the masonry beneath the insulation (see Fig. 4).

It is undeniable that neither the leaks nor the wind-driven rain exposure are evenly distributed over the building envelope. But the standard committee chose this simple one-dimensional approach as a method to consider the effects of complex bulk water penetration phenomena observed through forensic analysis. The rainwater leakage rate proposed in the standard is not meant to be a worst-case scenario. It is not based on field test results but on hygrothermal simulations [14] that showed that more than 1% of rainwater penetration may be detrimental for a large portion of existing wooden wall structures. A slightly more recent review, analysing data of leakage rates measured on different wall structures [15], confirmed the appropriateness of the ‘1% leakage’ in the ANSI/ASHRAE Standard 160. Therefore, the rationale of this standard was also adopted for the new WTA 6-2 guideline [16]. In the meantime, it appears that this leakage rate is rather conservative (on the safe side) when dealing with ventilated wall constructions [17].

### Air leakage

The convective moisture entry, due to imperfections in the vapour, and/or air, control layer is a multidimensional effect, which cannot be captured directly by a one-dimensional calculation. However, the application of multidimensional simulation tools hardly solves the problem, because the exact configuration of leakages is generally unknown, and the complexity of relevant flow paths is exceeding the capacity of most models. Therefore, the committee of the WTA 6-2 guideline decided to adopt an approach that does not simulate the flow itself but concentrates on the effects of vapour convection and subsequent condensation by introducing a moisture source inside the construction.

**Figure 6:**
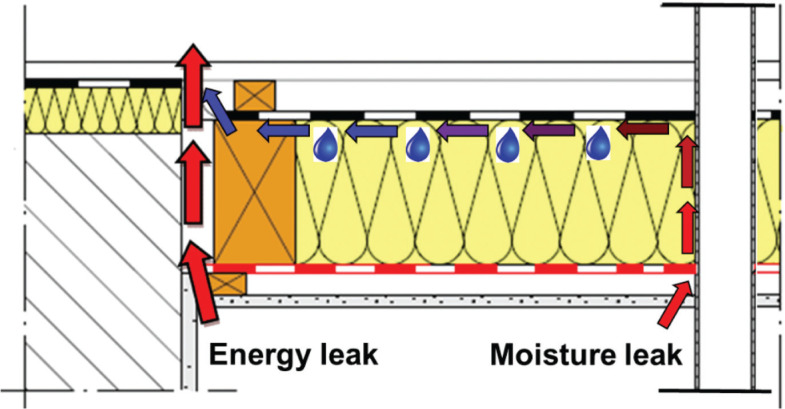
Indoor air leaking through a roof at joints and connections. If the flow path is short, it will be heated up by the air flow and only energy is lost. If the air flow creeps along the cold side of the structure before it finds its way out, its temperature may drop below the dewpoint of the indoor air and cause condensation.

Based on experimental results in TenWolde et al. [18], a simplified model to quantify the moisture sources due to vapour flow through the building envelope has been developed and checked for plausibility, for example, Künzel et al. [19]. The model assumes that vapour contained in the indoor air, penetrating the envelope via so-called moisture leaks, condenses at the cold side of the insulation (see Fig. 6). In contrast to energy leaks where the air remains warm because it flows in a direct way from the room towards the outside, moisture leaks are small and tortuous channels where the air flow is slow and cools down within the flow path.

These ‘moisture leaks’ probably represent less than 10% of all leaks in the building envelope. The position of the condensation plane needs to be selected by the user. Its temperature, governed by the transient boundary conditions, is simulated without taking the latent heat of condensation into account. The right choice of this position depends on the construction. It must be cold enough for condensation to occur and it must be easily accessible for the indoor air that has penetrated the interior lining or air barrier. Examples are the exterior sheathing of wood frame walls or roofs and the interface between the interior insulation and the original wall after thermal retrofits of plastered masonry structures. The convective moisture source is equal to the amount of condensate that forms when the indoor air temperature is cooled down to the temperature of the selected condensation plane in the building assembly. Any increase in sorption water content that could occur in reality by the temperature drop is neglected. In order to remain on the safe side, convective drying is excluded, that is, the moisture accumulated by air convection can only dry out by vapour diffusion or liquid transport.

Thus, the amount of condensation (moisture source *S*_CL_) which results from vapour convection at the selected condensation plane is determined for each time step according to the following equation:

(1) $$S_{CL}=k_{CL}\cdot (c_{i}-c_{sat,p})\cdot (P_{i}-P_{e})$$

where *S*_CL_ is the moisture source due to vapour convection through the component (kg/(m^2^h))

*k*_CL_ is the air permeance of the ‘moisture leaks’ of the component (m^3^/(m^2^·h·Pa))

*c*_i_ is the water vapour concentration of the indoor air (kg/m^3^)

*c*_sat,p_ is the water vapour saturation concentration at predefined plane p (kg/m^3^)

*P*_i_ – *P*_e_ is the air pressure difference over the considered envelope component (Pa)

The air pressure difference is assumed to be due to buoyancy effects and pressure differentials generated by ventilation systems. Wind pressure effects are disregarded here, because they are difficult to determine and do not act on the building envelope in a continuous or uniform manner. Moreover, wind pressure effects rarely follow a seasonal pattern, which means they will not only lead to wetting in winter but also to drying of the airflow path in summer.

Defining a representative component air permeance is challenging because it cannot be determined by simple blower-door tests. Such air tightness tests will only deliver the total air leakage rate without differentiating between energy and moisture leaks (see Fig. 5). Furthermore, as this one-dimensional approach is not a physical representation of the real phenomena, but rather a method to make the results of hygrothermal simulations more realistic, the choice of the component air permeance also defines the upper limit for installation deficiencies. This means that envelope components having a higher permeance than defined are not covered by this approach. Based on a study by Künzel et al. [19], the air permeance of the moisture leaks in flat roofs and stud walls has been set to 1.9×10^−6^ m^3^/(m^2^s·Pa) [0.007 m^3^/(m^2^h·Pa)] for envelope components installed according to best practice. Buildings with higher *k*_CL_ would represent malpractice. Building components that can handle the moisture loads due to air convection are well designed. Those that fail under these circumstances should either be redesigned, or special care must be taken during installation, which may include continuous moisture monitoring.

It should be noted that the choice of the component air permeance may have legal repercussions, because it allows to determine the cause of damage, which helps to disclose who may be responsible. If, for example, the hygrothermal analysis proves that a small unavoidable imperfection led to the failure of the system, it would signify inappropriate moisture control design. If, however, the analysis shows that the system is moisture tolerant and failure only occurred because the air permeability of the system must have exceeded the maximum leakage rate acceptable under best practice conditions, the installer would bear the responsibility. Hence, there should be broad consensus about the definition of the component air permeance limit. This limit could also be a function of climate, construction type and installation practice.

## European moisture control design standards and guidelines

Moisture control standards and guidelines evolved almost simultaneously in Europe and North America after World War II (WWII). In the beginning the main focus was preventing interior surface condensation, later followed by managing interstitial condensation. Even before that, driving rain protection had been common practice among builders mostly without specific standards to rely upon. Examples are cavity walls, brick walls with a continuous mortar layer between exterior and interior wythe and walls clad by shingles or slate at the exposed orientation. Modern lightweight blocks large enough for single layer masonry sped-up the construction process of external walls in Germany after WWII. However, in regions with high driving rain load, these external walls appeared to be prone to high moisture content resulting in durability problems and water stain at the interior surface [20]. The reason proved to be insufficient protection against rainwater being absorbed by the masonry through the external render. In contrast to traditional brick walls, the modern lightweight block walls did not have any mortar joints parallel to the façade. As these mortar joints act to some extent as capillary breaks for liquid water, their absence led to deeper water penetration during driving rain events. As consequence, new external rendering systems were developed that reduced the water absorption of the exposed wall while being vapour permeable enough to support the drying process [20]. Later, the criteria developed to characterise these so-called water repellent rendering systems were introduced into the German standard on moisture control. This means that the standard requires not only interstitial condensation calculations (dewpoint method) but also the assessment of the driving rain protection of external walls based on the local driving rain load and exposure situation. Alternatively, hygrothermal simulations may be performed, which include rain and wind data as explained below.

As climate and building traditions differ from country to country, there will be no set of rules that fits all constructions and climate zones. In Australia, for example, cavity masonry and timber framed construction methods were adopted from the UK. Due to Australia’s more temperate climates, there was no requirement for insulation in most jurisdictions prior to 2002. Therefore, it is not surprising that the first moisture control standards were developed by each country individually on a national basis. Later, some evaluation techniques such as the dewpoint method and hygrothermal simulation model requirements were harmonised by international standards. The development of the international standards and guidelines as well as their practical applicability is explained below. Afterwards, the basic principle of the recent German moisture control standard is described as an example for the national implementation of international provisions.

### EN ISO 13788 – Dewpoint calculation standard

Many architects and engineers still rely on simple steady-state tools to provide guidance that their constructions will be free of interstitial condensation based on EN ISO 13788 [21]. Looking at this standard more closely proves that there are many restrictions for its application and the outcome is not always on the safe side. If in doubt whether the steady-state method is applicable, EN ISO 13788 refers to more sophisticated evaluation tools. The *ASHRAE Handbook of Fundamentals* [1] goes a step further and states: ‘The dew-point method is not recommended as a sole basis for hygrothermal design of building envelope assemblies. ASHRAE Standard 160 is recommended to assist in hygrothermal analysis for design purposes’. ASHRAE Standard 160 specifies the application of hygrothermal simulation tools classified as type 4 in Hens and Janssens [22]. Originally, the steady-state calculation method in EN ISO 13788 was developed by Glaser [23] to determine the risk of interstitial condensation in the envelope of cold storage rooms. Later he transferred it to buildings made of lightweight components. In these cases, the method still works very well, provided driving rain is not a matter of concern. The method is very easy to understand and the solution to prevent interstitial condensation is simple: add a vapour barrier to the warm side and everything will be fine.

However, this simple solution has turned out to be the one of the biggest problems for the building industry, especially for the manufacturers of timber frame constructions. Having a vapour barrier at one side and a sealing membrane on the other side may be a recipe for disaster, because it means that any moisture getting in between will be trapped there for a long time. Practical experience has demonstrated, time and again, that moisture will find a way into building components even if metal foils are used as vapour barriers. The authors of EN ISO 13788 were aware of this problem and added a section on determining the drying potential of constructions having vapour retarding layers on both sides. In section 7 of the standard the user is asked to add 1 kg/m^2^ of water in the middle of a material layer that may get wet by accident. Afterwards the normal procedure of calculating interstitial condensation and drying by applying the standard monthly mean indoor and outdoor conditions is continued until all the water has dried out. The number of months necessary to achieve the dry state must be reported. If there is still moisture in the assembly after running through the whole set of boundary conditions 10 times (10-year period) the calculation should be stopped. In any case, the user must assess the risk posed by the local amounts of water in the assembly and the duration of the state of wetness in the different component layers. No recommendations are given on how the moisture risks should be evaluated.

The vagueness of condensation limits demonstrates the dilemma of the simple vapour diffusion calculation method in the standard. It calculates the theoretical amount of condensation in different layers or at interfaces, but it does not recommend any safe amount of interstitial condensation apart from stating that condensate on non-water-absorbing interfaces exceeding 200 g/m^2^ can run down (and may cause problems elsewhere in the component). In section 7, five times this amount is added, which is the absolute maximum that many national standards accept before warning of component failure. To be fair, establishing moisture content limits is also a challenge for more sophisticated methods such as hygrothermal simulation models. The main problem with calculating potential amounts of localised condensation is that many modern building materials do not really experience condensation because water vapour is absorbed and redistributed within the pore structure, as, for example, in capillary active insulation materials [24] such as mineral-based insulation panels or masonry blocks and rendering systems with high thermal resistance. Establishing a limit for condensation that happens only on paper respectively in the calculation tool makes little sense.

### EN 15026 – Hygrothermal simulation standard

Fully fledged hygrothermal simulation tools for building application appeared in the early 1980s, for example, Kiessl [25], and became more widely developed and applied in the 1990s. In 1998, the International Energy Agency (IEA) Annex 24 on heat, air and moisture transfer in insulated envelopes [22] compiled and classified the hygrothermal computer programs internationally employed (mostly by universities) at that time. Only half of the 29 programs evaluated, complied more or less with the requirements later established for hygrothermal simulation tools in EN 15026 [26]. Common exercises performed during the project demonstrated that simulation results were surprisingly close to each other and mostly matched experimental benchmarks.

This encouraging outcome and frustration with the dewpoint method among heritage restoration architects and forensic experts led to new efforts to push the acceptance of hygrothermal simulation results among design and construction professionals as an alternative to steady-state moisture control assessment methods. To ensure reproducible simulation results, new application standards for hygrothermal simulation tools had to be developed. The first guideline on moisture control analysis by hygrothermal simulation was issued in 2002 by the WTA (Wissenschaftlich-Technische Arbeitsgemeinschaft für Bauwerkserhaltung und Denkmalpflege), a European association dealing with preservation and renovation of heritage constructions and rehabilitation of the building stock [16]. Five years later the European Standard EN 15026 [26], which is largely based on the WTA guideline, was published. However, both documents did not contain any information on how to deal with small defects in the building envelope. As already mentioned above, this has been remedied in the new version of WTA 6-2 of 2014 by introducing an appropriate source term. It is expected that the EN 15026 will soon follow suit.

Parallel to the standard work in Europe, a slightly more comprehensive standard on moisture control design has been developed in North America (ANSI/ASHRAE Standard 160 from 2009, the original version of [9]). As a result of numerous damage cases linked to rainwater penetration into constructions with rendered facades, this standard has been the first that proposed the consideration of the effects of small leaks in the exterior finish of exposed walls. The standard is continuously updated with the most recent version being from 2021 [9].

Australia, being a late starter in this field, is currently experiencing significant mould and interstitial condensation in buildings designed and constructed over the last decade. Currently, Australia has no local national standards or guidelines and is looking to the experiences of the UK, the US and Germany to inform regulatory development.

### Boundary conditions

EN 15026 [26] specifies the heat and moisture transfer phenomena to be considered by hygrothermal simulation tools. It also recommends a choice of boundary conditions, which will not be repeated here, because national standards may endorse their own indoor and outdoor conditions. There are, however, some important remarks to be made concerning the selection of the outdoor climate data. The preferred choice in EN 15026 is a meteorological dataset for the location of the building that consists of hourly values for at least 10 consecutive years. While this is a very reasonable recommendation, it may be difficult to obtain such a complete 10-year dataset. As the climate data changes from year to year, the calculation results also vary accordingly. The subsequent scatter of yearly highs and lows in water content makes the evaluation of results more challenging. Convergence errors are less easy to detect and in the case of initial moisture uptake of the considered envelope component, the dynamic equilibrium is harder to define. Therefore, most users prefer to have only one representative meteorological dataset per location.

The second recommended choice of climate data in EN 15026 is the application of a design reference year that causes the most severe moisture conditions in an envelope component likely to occur every 10 years. However, the definition of most severe is ambiguous. Originally, it was meant to be the year with the one in 10 highest amounts of interstitial condensation in a reference component, that is, a rather cold year. However, it could also be a very warm year for a cold storage building or an air-conditioned building in a warmer climate. It could also be a year with a large amount of rain, if the reference wall construction is exposed to the main driving rain orientation and has a water absorbing exterior cladding. In Australia the El Niño-Southern Oscillation (ENSO) includes two cycles, namely El Niño and La Niña, with El Niño representing many months with warmer and drier conditions, whilst the La Niña period represents many months of wetter than average conditions. Even considering these variables, if interstitial condensation is expected to be the dominant failure criterion, employing such a severe meteorological dataset repeatedly to simulate the moisture behaviour over several years is generally discouraged because such a situation (one very severe year after the other) may occur only once in a century or millennium. Thus, well-proven constructions may fail the simulation test and only very costly or less environmentally friendly assemblies will pass.

Therefore, such a severe dataset representing a year that occurs only once in 10 years should be used only in combination with datasets of average years. However, most of the common average datasets have been constructed for building energy calculations, the so-called test reference years (TRY). They may or may not be appropriate for hygrothermal simulations, because they have not been designed to represent average moisture-related parameters such as air humidity or driving rain frequency. The best method to construct a representative dataset suitable for hygrothermal envelope simulations, the so-called moisture design reference years (MDRY) or hygrothermal reference years (HRY), is still a matter of debate. A feasible approach appears to be the selection of a meteorological dataset which if employed repeatedly arrives at equivalent results as the application of a dataset consisting of approximately 10 consecutive real years, as explained in Schöner and Zirkelbach [27]. This is in contrast to the TRY datasets that have been selected based on solar radiation and temperature.

It seems impossible to define an optimum approach to construct the most suitable HRY for all applications and construction types. There will always be different solutions depending on the problem to be investigated, for example, condensation, mould growth, rot, corrosion, frost damage or degradation due to hygrothermal stress and strain. Sometimes, there may even be a need for accelerating long-term hygrothermal phenomena to achieve critical conditions faster to save calculation time, as described in Tanaka and Zirkelbach [28]. However, in many design applications, the potentially critical damage process is unknown before the simulation starts. Therefore, it makes sense to begin with an average HRY and resort to more critical datasets if the results indicate that there may be problems during more severe weather conditions, for example, water content, temperature or humidity within the assembly getting close to safe limits.

More important than analysing the suitability of existing meteorological datasets belonging to one particular climate zone can be the assessment of the local situation of the building. If the considered building is located at an altitude well above the weather station, or close to a lake or in a shaded valley, the environmental conditions may differ significantly from the location of the weather station. In such a case the meteorological dataset should be adapted to account for the local situation, for example, as explained in Wang et al. [29]. There are also various models that project future climate conditions; however, this is usually beyond the scope of normal design tasks. Accounting for changing climate conditions may be beneficial for new constructions that are meant to last, but it is even more important to assess their impact on our heritage buildings. For more information, please refer to Leissner et al. [30].

In conclusion, for most applications the best choice of outdoor climate data appears to be those that represent average weather conditions at the building site. As there is a tendency to add a safety factor to the interior climate, for example, by defining boundary conditions that represent the upper limit of existing residential indoor climate conditions, there is no need to apply a second safety factor to the outdoor climate. Doing this may not only be too conservative, that is, constructions that work well in practice may fail in the simulation. It can also leave other problems undetected, for example, summer condensation if severely cold meteorological datasets are used. It is, however, essential that the selected meteorological dataset contains all relevant climate parameters as measured hourly values. Data generated by models from multi-hour, daily or monthly mean values may incur erroneous simulation results especially if the envelope component is exposed to wind-driven rain loads [31].

### Evaluation of simulation results

The section on result interpretation in EN 15026 is rather short and leaves room for more specific national regulations. It proposes only the following four items of which at least one should be considered to establish the relevant pass criteria for envelope components:

- Compare the resulting hygrothermal conditions with specified limits.
- Check the risk of moisture accumulation by comparing the total moisture content after one cycle with the initial condition.
- Evaluate the moisture tolerance of the construction (drying potential).
- Feed the transient results into a post-process model (e.g., for mould or algae growth, rot, corrosion).

These criteria do not represent clear guidance for practitioners. Therefore, experience and good professional judgement would be needed to arrive at a robust decision. Checking the risk of moisture accumulation is probably the easiest task; however, one cycle may not be enough to come to the right conclusion. Depending on the selected initial conditions and the type of construction, several annual cycles may be required to confirm the long-term performance. WTA 6-2 [15] assumes that the dynamic equilibrium is reached when the change in total water content after two subsequent cycles is less than 1%. Comparing the simulation results with specified limits is more challenging unless they are given in material specifications or guidelines. Single value limits may be misleading because degradation risks due to elevated water content or humidity often depend on the temperature range. For example, mould growth, rot and corrosion are accelerated by higher temperatures. Thus, it is not sufficient to specify safe limits for water content or humidity, also the local temperature and possibly other parameters such as pH, pollutant concentration or particular material characteristics may have to be considered. This is the reason why the so-called post-process models have become increasingly important.

However, apart from mould growth models, there are currently very few post-process models available, such as the corrosion model in Marra et al. [32], which had been developed to estimate the corrosion progress of iron inserts in historic mortars. As mould fungi and their spores may affect human health, there have been considerable research efforts to predict the risk of mould growth on building component surfaces or interfaces. ASHRAE Standard 160 [9] now recommends the use of the empirical mould growth model from Viitanen and Salonvaara [33]. The first version of this standard from 2009 still had a rather simplified mould growth prevention criterion. It specified the following limits for the 30-day running average of material interface conditions: within the temperature range between 5°C and 40°C, the surface humidity had to remain below 80% RH. As this criterion proved to be overly conservative, it was replaced by the above-mentioned mould growth model in the version of 2016.

Researchers have also developed other mould growth models, for example, the semi-physical model of Sedlbauer [34]. This model was originally designed to predict the mould growth risk on surfaces facing the interior living spaces. However, it has also been used to predict mould growth at material interfaces of timber frame constructions, for example, Brambilla and Gasparri [35]. In contrast to the Viitanen model, the Sedlbauer model only accounts for mould growth under favourable condition and does not have a decline function when ambient conditions are very unfavourable, for example, very dry or cold. Therefore, it may signal continuous growth over long periods while, in reality, the intermittent decline helps to keep visible growth at bay. Both models calculate growth as functions of transient humidity and temperature conditions.

**Figure 7:**
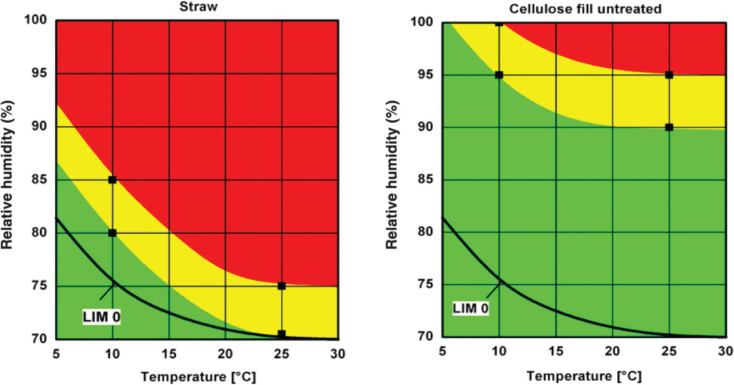
Mould growth sensitivity of two building insulation materials illustrated by the RH–temperature range of growth probability. Green indicates no growth risk, yellow low growth risk and red high growth risk [36].

Another important parameter is the material’s sensitivity to mould growth, which mainly depends on the presence of nutrients (promoting growth) or preservatives (inhibiting growth). Figure 7 shows the so-called isopleths (lines of equal growth rate in an RH over temperature diagram) described in Sedlbauer [34] for straw and untreated cellulose fibre insulation determined by climate chamber tests [36]. There is a clear difference in mould growth risk. While growth on straw can be expected to start above 85% RH at 10°C and around 75% RH at 25°C, cellulose fibres are much more resistant to mould and will not show any growth below 95% RH. This important difference shows that is essential to obtain information about the mould growth sensitivity of building materials employed in humid environments. While there is no doubt that the current mould growth models represent a real improvement compared to the simple growth limit of the monthly running average of 80% RH, the different impact factors, such as substrate characteristics, duration of favourable and unfavourable or even lethal surface conditions, are affected by considerable uncertainties. Therefore, more experimental research and continuous model improvement are still required [37].

Summarising the current state-of-the-art concerning moisture control design by hygrothermal simulation: we have the necessary models and simulation tools; we know how to obtain or generate the input data; and we have a fair idea of how the simulation results should be evaluated. Therefore, we can expect that building science experts will generally be able to deliver an adequate hygrothermal analysis and propose working building envelope solutions. However, inexperienced architects may find it difficult to employ hygrothermal simulation tools confidently and the effort involved in a detailed hygrothermal analysis is often not rewarded by the client. Therefore, there must be a more flexible approach to moisture control for the design and construction professions. It should include a list of deemed to satisfy elemental construction methods subject to some restrictions, for example, indoor climate conditions representative for residential and office buildings, and normal exposure conditions (e.g., excluding locations high up in the mountains). This list should contain assemblies that already have a long track record and possibly also those that have been studied extensively by field tests or hygrothermal simulations. As the Glaser method is well established and may give useful results for some construction types, it could also be part of the analysis if the limits of this method are clearly observed. The German moisture control standard for building envelope components DIN 4108-3 updated in October 2018 [38] may serve as an example for such a flexible approach, therefore its general outline is presented here.

### DIN 4108-3 moisture control standard offering a three-pathway approach

**Figure 8:**
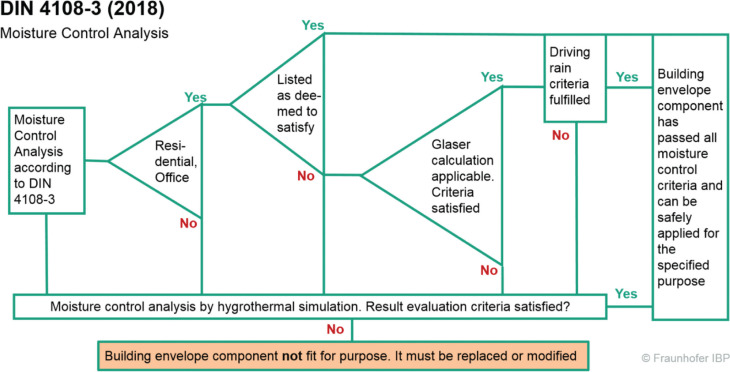
Flow chart explaining the preconditions for the moisture control assessment options in DIN 4108-3 [38].

Due to a history of severe building failures caused by moisture problems, German builders, material manufacturers and forensic experts have been searching for better moisture control design guidelines, which is not an easy task, considering the nature of the very conservative construction sector. As a compromise between ‘accurate but complicated’ and ‘easy but oversimplified’, the following three-pathway approach has been developed. The first option is to select a wall or roof assembly from a list of deemed to satisfy (DTS) constructions included in the standard. The second option represents the assessment by a steady-state dewpoint calculation with fixed boundary conditions. However, there are a number of restrictions that have to be observed. Constructions that must not be evaluated by the dewpoint method include, for example, vegetated or ballasted flat roofs and those shaded by elevated panels, interior insulation systems with *R* > 1.0 m^2^ K/W, and constructions in contact with the ground. Most importantly, pathway one and two may only be applied for envelope components of residential buildings or those that have a similar indoor climate (e.g., offices, shops, etc.) and no air-conditioning. In all other cases, pathway three applies, that is, the designer has to perform a hygrothermal simulation according to appendix D of the standard. This appendix references EN 15026 [26] and WTA 6-2 [16] as well as some other standards and guidelines dealing with the evaluation of hygrothermal simulation results, for example, mould risk evaluation. A flow chart showing the preconditions for selecting one of the options is depicted in Fig. 8.

### Further national guidelines dealing with moisture control design by hygrothermal simulation

Apart from the standards and guidelines in North America [9] and Germany [38] already covered, there is a trend to update moisture control recommendations around the world. The French PACTE (Programme d’Action pour la qualité de la Construction et Transition Energétique) has recently issued a guide that specifies the input parameters for assessing wall components for French climate zones by hygrothermal simulation [39]. It includes the consideration of construction imperfections and potential moisture sources due to indoor air infiltration or rainwater penetration.

In the UK, there is BS 5250 [40], which has had several versions since 1975, with the latest version being released in July 2021. In New Zealand, E3 Internal Moisture for new buildings was first published in 1992. Since then, there have been several amendments with the latest changes being planned for review and adoption in 2021/2022. Whilst Australia has had a very general condensation handbook since 2014, which has not provided adequate or specific guidance, included its first clause regarding moisture and condensation within the Health and Amenity section of the nation building regulations in 2019. However, the regulation only applied to stand-alone and multi-residential buildings in Australia’s cooler climates. It is hoped that some requirements will be extended to encompass most Australian climates in 2022. The 2022 update will reference AIRAH DA-07 [41], which is an Australian version of ASHRAE 160. Within Australia, all levels of the design and construction professions continue to request the government provide climatically specific floor, wall and roof system guidance.

These examples are far from being complete, but they show the growing importance of hygrothermal simulation models for energy efficient and durable building design. They also prove that the days of simple steady-state methods are numbered, because their outcomes may be misleading, resulting in damage, premature degradation or even unhealthy indoor conditions.

## Conclusions and further research needs

Hygrothermal simulation models have become essential to advance our skills in sustainable and resilient building design. However, their application only makes sense for building scientists or design and construction professionals with model application experience. Therefore, a more flexible approach including simplified evaluation methods may help to bridge the gap between science and practice.

Practical experience has proven repeatedly that imperfections and small leakages in the building envelope are unavoidable and must be accounted for. This is a new paradigm and must be included in all types of analysis. This is probably easier to achieve in simulation models, but it could also be transferred to simpler methods. The development of new approaches that can represent real life impacts caused by imperfections is a challenge, because it depends on the question ‘what is unavoidable?’. Ultimately, the definition of unavoidable moisture sources will result in a clear delineation between design and installation failures. This will also result in more moisture tolerant building component design. As a great deal of money is involved in this important classification, the discussions between design and construction professionals (architects and trades) will probably intensify in the future, but any progress in this field will be beneficial for the sustainability and long-term performance of our buildings.

Quantifying the impact of imperfections is essential and certainly requires further research and field surveys. However, solutions that enhance the drying potential of envelope components, such as the application of humidity controlled, variable resistance vapour retarders or programmable material layers, should be further investigated. Apart from selecting adequate moisture control characteristics for these functional layers, their long-term performance is of great relevance. The German Technical Approvals for humidity-controlled vapour retarders require cup tests of the new and the artificially aged membranes so as to exclude harmful degradation of the variable vapour diffusion performance. Generally, natural ageing of building materials and the impact on their hygrothermal and mechanical properties have only been investigated anecdotally, which means there is a need for more rigorous research. Similarly, questions are being asked about the published vapour resistivity properties of construction materials, as most are defined based on wet or dry cup tests at 23°C/50% RH. In reality, they experience varying temperatures and relative humidity conditions within the external envelope, and tests have identified non-linear relationships between relative humidity and vapour resistance [42].

In general, there is a demand for developing post-processing tools that can model material degradation as function of ambient conditions and hygrothermal loads. Besides further refinements to mould growth models, this includes approaches to assess the likelihood or progress of corrosion, rot of bio-based materials, frost damage, cracking due to hygrothermal stress/strain or degradation due to coinciding temperature and moisture peaks. The last effect, the combination of local heat and moisture load maxima or minima, appears to be a driving force for physical or chemical material ageing.

Keeping building materials dry has always been the aim of moisture protection measures. However, the growing emergence of more sustainable bio-based materials requires even stricter moisture limits than in the past. It is a well-known fact that timber constructions and wood-based building products are more moisture susceptible than their conventional mineral or polymeric peers. Therefore, improved moisture control strategies must be developed. However, today we are facing an even more challenging situation. As timber prices are rising due to limited resources, replacements made from other plant fibres or agricultural waste are being developed. Many of these composite products are more moisture susceptible than wood-based products, which means they grow mould more readily and are less resistant to rot. Preventive measures that do not include potentially toxic chemicals are required. Safe alternatives may involve bio-based materials with mineral compounds and binders as well as compositions of moisture susceptible and resistant materials responding to the microclimate in different building assemblies.

## Outlook

Within all developed and developing nations, the option to develop and add more and more moisture and mould resilient construction systems to the DTS lists within national Building Regulations and Standards and Guidelines, by performing hygrothermal simulation studies, has motivated manufacturers and trade associations to get their products tested and listed, so as to facilitate specification and selection by design and construction professionals. While this development has been anticipated and desired, it also reinforces the moisture control ranking of being the least important design step coming at the end of all other design issues. This means that the architect just looks at the DTS list and basically modifies the envelope design only marginally to adapt it to the requirements, for example, by just replacing the vapour retarder respectively the WRB (water-resistive barrier) or by adding a ventilation gap. This simplifies the design process, but it does not guarantee the optimum design solution.

**Figure 9:**
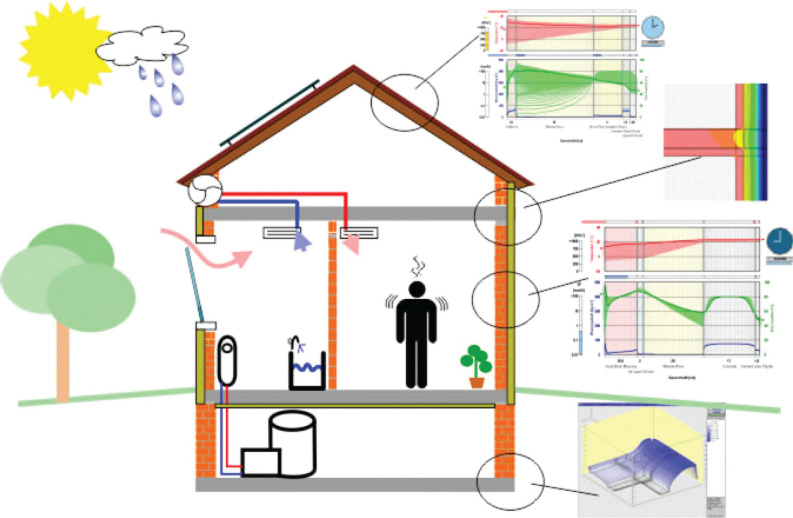
Schematic representation of building simulation models including not only thermal but also moisture transfer simulations for all relevant envelope components including transient thermal bridge calculations and considerations of transient HVAC performance as well as building operation modes and the behaviour of occupants [43]. The figure demonstrates the impacts of human activity and HVAC systems on the indoor climate and the hygrothermal interactions with the different envelope component simulations. Thus, the moisture buffering effect of the building envelope and the hygrothermal performance of envelope components can be accurately evaluated.

A better solution would be to make moisture control concerns part of the integrated design process. This could be done by adding moisture transfer approaches to the standard thermal calculation methods in building energy simulation tools as demonstrated in Fig. 9. This would allow design professionals to incorporate moisture retention phenomena together with the thermal inertia of building envelope components. At the same time, HVAC design and ventilation strategies could be optimised based on more realistic indoor humidity levels, which are known to be strongly influenced by moisture buffering effects of envelope components. Both of these principles fit well with the increasingly stringent requirements for building energy efficiency which require the considered design of both the external envelope and HVAC.

In addition to the benefits stated above, accounting for the humidity uptake or release of the building envelope (hygrothermal buffering), reproduces the transient indoor humidity conditions in a realistic way. Thus, the risk of moisture problems (condensation, mould growth, warping of wooden flooring) due to the increased presence of surface cooling systems, for example, chilled ceilings or floors, running during hot summers can be appropriately assessed. These cooling systems, which do not have the capacity to dehumidify the air, are on the rise in Central Europe, the US and Australia. In many new buildings, heat pumps that can also run in reverse mode are increasingly replacing gas boilers as heating systems. Furthermore, the situation of new constructions which contain considerable amounts of construction moisture, due to wet weather during construction, or material transport, may be analysed.

Last but not least, the more unstable renewable energy supply will put the focus on thermal storage capacities in buildings. This will probably increase the demand for thermal component activation (e.g., concrete core activation), envelope components containing phase change materials and new components with integrated heating and cooling systems. However, the thermal activation will always result in some form of moisture movement, which can only be accurately investigated with appropriate hygrothermal building simulation tools. Moisture transfer effects related to heat storage processes may help to stabilise the indoor climate conditions, but they can also be counterproductive, for example, if the surface humidity of some envelope components exceeds safe limits.
